# A declining pattern of malaria prevalence in Asendabo Health Center Jimma zone, Southwest Ethiopia

**DOI:** 10.1186/s13104-019-4329-6

**Published:** 2019-05-27

**Authors:** Abdurazak Jemal, Tsige Ketema

**Affiliations:** Department of Biology, College of Natural Sciences, Jimma University, Jimma, Ethiopia

**Keywords:** Malaria, Prevalence, *P. falciparum*, *P. vivax*, Seasonal variation

## Abstract

**Objective:**

To assess the status of malaria prevalence in one of the malaria endemic areas of Ethiopia.

**Results:**

A 10-year report of malaria cases were obtained from Asendabo Health Center, Jimma zone, Southwest Ethiopia. Following a retrospective study design, data of 68, 421 febrile patients diagnosed and treated in the health center were included in the study. The year with the highest prevalence rate (34.9%) was 2010, whereas the lowest was 2016 (0.62%). The number of diagnosed malaria cases from September to November were significantly higher (P = 0.023, n = 6336, 46.5%) than in other months. *Plasmodium falciparum* (52.1%, n = 7087) and *Plasmodium vivax* (44.2%, n = 6009) were the two principal plasmodium species accountable for malaria infections in the study area. The current study is a supportive evidence for the reduction of malaria prevalence in malaria endemic areas of Ethiopia.

**Electronic supplementary material:**

The online version of this article (10.1186/s13104-019-4329-6) contains supplementary material, which is available to authorized users.

## Introduction

Malaria is one of the top public health concerns of the nations in tropical and sub-tropical regions of the world. It is a major cause of morbidity and mortality in Africa [1]. Reports in 2005, showed that global estimate of deaths that occurred annually from malaria was 1–3 million [2]. However, a recent report of World Health Organization (WHO) showed a significant reduction of malaria associated deaths to 584,000 globally in 2013 [3]. Other reports by WHO has also indicated the reduction of malaria-related deaths among African children since 2000 [4].

In Ethiopia malaria is still one of the major health problems in some parts of the country. According to the Federal Ministry of Health (FMoH) report, about 60% of the country’s populations live in malaria endemic areas [5]. To create malaria-free nation, government has set a big goal of eliminating malaria by 2020 [5]. To achieve the set goal, all concerned bodies in the country have shown a strong commitment. Hence, the country has been able to achieved about 50% overall malaria reduction goal by 2015 [6]. This remarkable achievement was attained mainly due to the implementation of intensive interventional strategies such as indoor residual spraying, use of bed nets, and combination chemotherapy [7]. However, in some endemic areas of the same country, this reduction has not yet been achieved and even it is a major cause of illness and death [8]. One of the regions that have so far achieved remarkable malaria burden reduction is Oromia [9]. Jimma zone is one of malaria-endemic areas in the region and Asendabo is one of malaria endemic districts found in the zone (https://en.wikipedia.org/wiki/Jimma_Zone). In the district, intensive malaria interventional approaches have been executed for a long time. Thus, the current study was designed to assess the status of malaria prevalence in this district and draw the experience that could be shared by other similar districts.

## Main text

### Methods

#### Study area

The study was conducted in Asendabo district, located at 303 km southwest of Ethiopia. This district is one of the malaria endemic areas in the zone and received special attention from national and regional governments, NGOs and public institutions such as Jimma University, Center for tropical and infectious disease on malaria prevention and control campaigns [personal communication]. The district has only one health center that serves about a population of 33,981 (unpublished data from zonal health office). Malaria is one of the major health problems of the district. The two main plasmodium species: *P. vivax* and *P. falciparum* are responsible for malaria infection and *Anopheles arabiensis* is known as principal vector transmitting malaria in the district.

#### Study design and data collection process

A retrospective study was conducted to determine the prevalence of malaria by reviewing febrile patients’ medical record at Asendabo Health Center from September 2007 to August, 2017 (10 year period). Patients were diagnosed and treated following a standard operating procedure for malaria diagnosis. Peripheral blood smear examination was used to confirm malaria infection as per the recommendation of WHO. All the variables recoded and documented for each patients such as Age in range, sex, blood film status (+ve and −ve), plasmodium species (*P. falciparum, P. vivax* and mixed infection), and data of diagnosis on the weekly malaria reporting format were considered in the study.

#### Data analysis

Data was checked for correctness, and analyzed using SPSS (Armonk, NY: IBM Corp) version 20.0 software. Descriptive statistical tests were used for analysis of malaria prevalence, seasonal variation and demographic data. A One sample T-test and Relative Risk test were statistical tools used to analyze differences between means of variables and to show relative risk of different groups to malaria respectively. Significance level was considered at confidence interval (CI) of 95%.

### Results

#### Trends of malaria prevalence

In the current study data of 68,421 febrile patients diagnosed and treated in the health center over the 10 years were considered. About 13, 624 of them were malaria positive. This showed an aggregate malaria prevalence of 20.7% (95% CI 20.37–20.99) among febrile patients. From the positive patients, 52.5% (n = 7138, 95% CI 51.32–53.65) were males and 47.5% (n = 6457, 95% CI 46.28 to 48.73) were females. Prevalence of malaria among biologically risked group, children < 5 years, was 24.4% (n = 3315, 95% CI 22.91–25.86) (Table 1). Although the proportion of malaria positive children was large, their relative risk to acquire the infection compared to the adult’s (RR = 0.77, 95% CI 0.75–0.803) was significantly low.

**Table 1:** Trend showing 10 years malaria prevalence in Asendabo Health Center (2007–2016)

Year	Total examined	Malaria positive	Malaria prevalence	Patients in sex		<5 years (%)
				Male (%)	Female (%)	
2007	6497	1814	27.9	953 (52.5)	861 (47.5)	392 (21.6)
2008	6506	1802	27.7	910 (50.5)	892 (49.5)	375 (20.8)
2009	7266	2104	28.9	1058 (50.3)	1046 (49.7)	617 (29.3)
2010	14,235	4963	34.9	2670 (53.8)	2293 (46.2)	1205 (24.3)
2011	9095	1916	21.1	1020 (53.2)	896 (46.8)	452 (23.6)
2012	7218	770	10.7	401 (52.1)	369 (47.9)	211 (27.4)
2013	4578	137	2.99	76 (55.5)	61 (44.5)	35 (25.5)
2014	3654	38	1.04	21 (55.3)	17 (44.7)	6 (15.8)
2015	3879	33	0.85	15 (45.5)	18 (54.5)	13 (39.4)
2016	2874	18	0.62	14 (77.7)	4 (22.2)	9 (50)
2017	2619	29	1.1	18 (62.1)	11 (37.9)	10 (34.5)
Total	65,802	13,595	20.7	7138 (52.5)	6457 (47.5)	3315 (24.4)

**Fig. 1:**
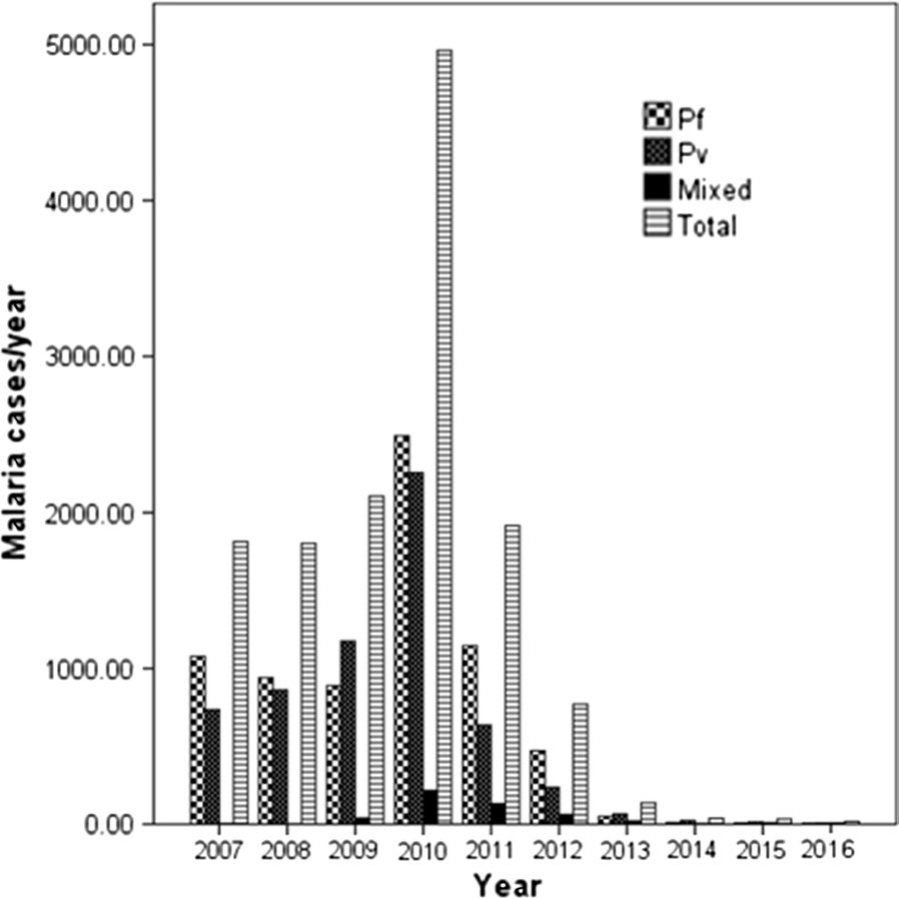
Plasmodium species accountable for malaria infection in Asendabo Health Center (2007–2016). *Pf*: *Plasmodium falciparum*; *Pv*: *Plasmodium vivax*; *Mixed*: *infection with both P.f and P.v*

Although the cumulative 10 years prevalence of malaria in febrile patients shown high, the trend in the study area has a declining pattern, from 27.9% in 2007 to 0.62% in 2016. The highest malaria prevalence among febrile patients (34.9%, n = 4963) was documented in the year 2010 and followed by 2009 (29.4%, n = 2104). The recent year’s assessment showed that, starting from 2013 to 2016, the prevalence was drastically reduced from two digits to 0.6% (1.375 averages) (Table 1). In 2016/2017, from September, 2016 to August, 2017, a total of 2619 febrile cases were examined for malaria infection. Only 29 (1.1%) of them were malaria positive. This showed that the declining pattern was persistent with slight increment from 0.6% (18/2874) in 2015/16 to 1.1% (29/2619) in 2016/2017, but not significantly different (P = 0.056). Majority of the positive cases (n = 17, 58.6%) were registered in a month (August). Among the total 29 positive patients observed, n = 10 were children < 5 years. The two Plasmodium species were accountable for malaria infections in the study area. About 52.1% (n = 7087) and 44.2% (n = 6508) malaria cases were infected with *Plasmodium falciparum* and *P. vivax* respectively. The remaining 7.7% were due to mixed (*P. falciparum* and *P. vivax*) infection (Fig. 1).

#### Seasonal variation and prevalence of malaria

From the analysis made to assess role of seasonal variations on the prevalence of malaria among febrile patients, it was observed that Autumn season (from September to November in Ethiopia) was found as malaria peak season in the study area. In other seasons such as Spring, Winter and Summer, the prevalence pattern seems similar. Number of malaria diagnosed patients in Autumn (46.5%, n = 6336) was significantly higher (P = 0.023) than other seasons. Seasonal variation showed effects on the type of plasmodium parasite infection. While *P. falciparum* and *P. vivax* were the two dominant parasite, prevalence of *P. vivax* among febrile patients was significantly higher (P = 0.042) compared to *P. falciparum* during Winter season (December to February in Ethiopia) than other seasons (Additional file 1).

### Discussion

In the current study the aggregate 10 years malaria prevalence observed was 20.7%. The prevalence rate was varies from 34.9% prevalence in 2010 to 0.62% in 2016. Similar to the situation in other developing countries, malaria was the major public health problem in the study area. However, recently due to a strong commitment shown by concerned bodies, the prevalence of malaria is radically reducing in most parts of the country [6]. Thus, the observed significant reduction in malaria prevalence may suggest the effectiveness of the interventional strategies adopted in the study area.

While the decreasing pattern of malaria prevalence throughout the 10 year was consistent, the fact that slight increment observed in the recent year (2016/17) has indication of possibility of malaria resurgence in the study area. Although it is possible to achieve remarkable malaria prevalence reduction, complete elimination of the infection is impossible [10, 11]. This could be due to the presence of persistent residual infection transmission and the biological nature of the parasite [12]. Thus, close monitoring and surveillance of the interventional strategies and the infection pattern is very important in such locality.

One of the determinant factors for malaria transmission is seasonal variation. Temperature and humidity are variables that govern plasmodium parasite growth in the vector body, and the vector development in the environment. Thus, optimum environmental condition is very important for the disease to transmit. Autumn season (after heavy rain) was found to be a malaria peak transmission season in the study area [13]. This finding is in agreement with a report of Jamil and Khan [14] from Pakistani and Woyessa et al. [15] from Butajira-Ethiopia, where prevalence of *P. falciparum* malaria reached its highest frequency after heavy rain.

One of the factors that might have contributed to the successful malaria reduction in the country is, direct involvement of health extension workers (HEWs) in malaria control and prevention campaign [16]. Their major role is enhancing awareness of the community towards malaria infection, its transmission, and on how to use of different prevention methods in malaria endemic areas [16, 17]. These workers spend 75% of their time visiting families in their homes and performing outreach activities in the community and distribute artemisinin-based combination therapy (ACT) to the needy community [18]. Thus, presence of HEWs is one of the important factors for early observation and reporting of malaria cases, for further diagnosis at the nearby health facilities, and to get treatment with appropriate antimalarial drugs [19]. This could be considered as best practice to be shared by other similar people living in malaria endemic areas in the country and beyond, with similar set-up. In general the use of bed net, IRS, combination therapy and contribution of different stakeholders including HEW might have a great role for the declining of malaria infection in the study area [20].

### Conclusion

The current study provides supportive evidence for the reduction of malaria prevalence in Jimma zone, Asendabo district. This could serve as a sign for the rise in commitment of all stakeholders to eliminate malaria from the country. The observed declining of malaria prevalence among febrile patients in this study is a promising outcome which could be cascaded to other malaria endemic areas.

## Limitation

This study was delimited to only one district. More concrete evidences could have been generated had it been included more malaria-endemic areas in the zone. In addition, only those patients seeking medication and visited the health center during the study period were included in the study. Data of other suspected malaria patients treated at home or used other traditional medication was not included in the study.

## Additional file

**Additional file 1: Figure S1.** Prevalence of plasmodium species with respect to seasonal variation at Asendabo Health Center, (January, 2007–August, 2016).

## Data Availability

The datasets supporting the conclusions of this article are included within the manuscript.

## Abbreviations

ACT: artemisinin combination therapy
DDT: dichlorodiphenyltrichloroethane
FDRE: Federal Democratic Republic of Ethiopia
HEW: health extension workers
IRS: indoor residual spraying
LLITN: long-lasting insecticide treated nets
UNICEF: United Nations Children’s Fund
WHO: World Health Organization
