# Supplementary material for: A declining pattern of malaria prevalence in Asendabo Health Center Jimma zone, Southwest Ethiopia
Source: BMC Res Notes. 2019 May 27;12:290. doi: 10.1186/s13104-019-4329-6 (PMC6537395; doi:10.1186/s13104-019-4329-6)
Supplement: Supplementary file 1 — Additional file 1: Figure S1. Prevalence of plasmodium species with respect to seasonal variation at Asendabo Health Center, (January, 2007–August, 2016). [file 13104_2019_4329_MOESM1_ESM.docx]

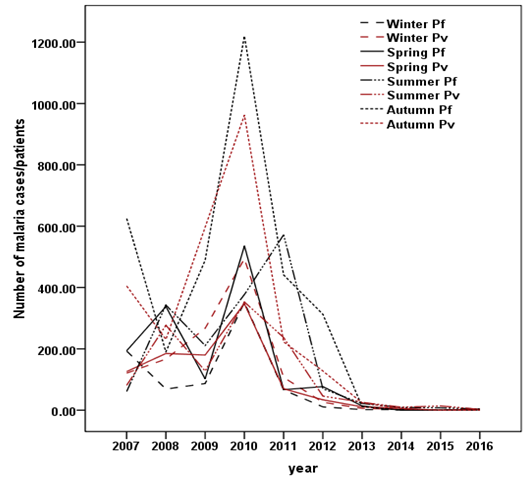


Note: Pf = Plasmodium falciparum, Pv = Plasmodium vivax,

Figure S1 Prevalence of plasmodium species with respect to seasonal variation at Asendabo Health Center, (January, 2007-August, 2016)
